# Inclusive growth: A dataset on key and institutional foundations for inclusive development of Russian regions

**DOI:** 10.1016/j.dib.2019.103864

**Published:** 2019-03-20

**Authors:** Rustam Ilfarovich Sharafutdinov, Elvir Munirovich Akhmetshin, Aleksandra Grigorievna Polyakova, Vladislav Olegovych Gerasimov, Raisa Nikolaevna Shpakova, Mariya Vladimirovna Mikhailova

**Affiliations:** a Academy of Sciences of the Republic of Tatarstan, Center of Advanced Economic Research, Kazan, Russian Federation; b Plekhanov Russian University of Economics, Industrial University of Tyumen, Russian Federation; с Financial University under the Government of the Russian Federation, Moscow, Russian Federation; d Plekhanov Russian University of Economics, Moscow, Russian Federation; e Kazan Federal University, Naberezhnye Chelny Institute of KFU, Naberezhnye Chelny, Russian Federation; f Moscow State Institute of International Relations (MGIMO), Moscow, Russian Federation; g I.M. Sechenov First Moscow State Medical University, Moscow, Russian Federation

## Abstract

This article presents a dataset for calculating the index of inclusive growth in the regions of the Russian Federation, estimated on key and institutional foundations of the performance of inclusive development. The authors of the research used the methodology of the World Economic Bank and the World Economic Forum, based on a comparative analysis of key and institutional indicators of the performance of territorial entities, which they adapted to the socio-economic features of the regional division of Russia. For the purpose of formation of a dataset was executed assessment of the inclusive growth index for 26 regions of the Russian Federation, it allowed to define the strengths and weaknesses of the inclusive development of each region of the Russian Federation. The dataset can be useful in the formation of strategic programs of inclusive development.

**Table:** Specifications table

Subject area	*Economics*
More specific subject area	*Income distribution between generations; total human capital; socio-economic development.*
Type of data	*Table, image, figure*
How data was acquired	The dataset on inclusive growth indices of the Russian Federation regions was calculated based on the analysis of information databases and the processing of queries received from official sources of state and regional statistics of the Russian Federation socio-economic development, formulas and methodologies proposed by the World Economic Bank specialists for calculating the inclusive development index. The obtained data was analyzed, processed, aggregated and structured under the methodology for calculating inclusive growth and development. For the formation of the final dataset, open sources were used: the database of the Unified Interdepartmental Statistical Information System (UISIS); Federal State Statistics Service, Statistics Map of the Russian Federation, as well as the results of previous studies on this topic.
Data format	*Analyzed, evaluated.*
Experimental factors	*Socio-economic stratification.* *At the first stage, the data necessary for finding key indicators of inclusive development were collected, which were then processed and calculated in sequence, with the addition and adaptation of a number of indicators for each specific region of Russia. At the second stage, the institutional foundations of inclusive development were assembled - education and infrastructure, which form the components of human potential, and were calculated according to a number of indicators adapted to the peculiarities of the socio-economic development of the regions of the Russian Federation.*
Experimental features	*Indicators of inclusive growth: key and institutional ones, and the degree of their development in the regions of the Russian Federation*
Data source location	*26 regions of the Russian Federation: Krasnoyarsk Territory, Kamchatka Territory, Arkhangelsk Region, Murmansk Region, Tatarstan Republic, Leningrad Region, Belgorod Region, Khabarovsk Territory, Tomsk Region, Sverdlovsk Region, Moscow Region, Irkutsk Region, Perm Territory, Orenburg Region, Samara Region, Vologda Region, Primorye Territory, Novgorod Region, Novosibirsk Region, Krasnodar Territory, Kaliningrad Region, Lipetsk Region, Kaluga Region, Yaroslavl Region, Bashkortostan Republic, Nizhni Novgorod Region.*
Data accessibility	Data is with this article.
Related research article	Sharafutdinov, R. I., Izmailova, D. O., Akhmetshin, E. M. (2018). Studies in National Key Performance Indicators of Inclusive Growth and Development of the Regions of the Russian Federation. Theoretical and Applied Economics, 3, 118–134. https://doi.org/10.25136/2409-8647.2018.3.27061. URL: http://e-notabene.ru/etc/article_27061.html[1]

**Table:** 

**Value of the data** • Final data on inclusive development helps to take a fresh look at the problems of the development of the theory of regional economics on based on expanded the theoretical grounds and methodological approaches to assessing inclusive growth and development of the Russian Federation regions. • The dataset can be used in the formulation of regional programs for socio-economic development, which in the long term will contribute to an increase in general well-being, equalization of incomes of the population and reduction of social inequality. • Key national inclusive development performance indicators adapted in accordance with the methodology of the World Bank and the regional characteristics of the territorial entities of Russia, make it possible to use them in assessing other Russian Federation regions.

## 1 Data

**Fig. 1:**
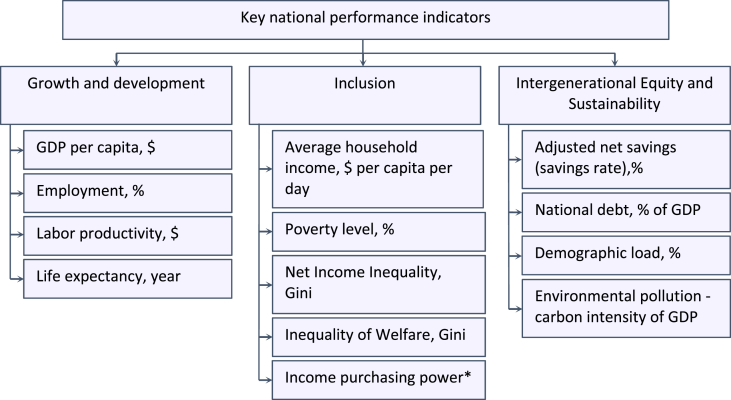
Key indicators of the performance of inclusive development [4].

Key indicators of inclusive development performance used to generate the data are divided into three categories (Fig. 1).

Growth and development is characterized by such indicators and data as: GDP (GRP) per capita (gross regional product, divided by the average annual number), labor productivity, determined from the gross regional product divided by the number of economically active population, life expectancy and employment, which is the ratio of the employed population to the total population.

The dataset presenting “Inclusion” is characterized by indicators reflecting the overall level of equity in the distribution of income among citizens, these include: Gini income ratio, poverty level, wealth inequality, average income per day. The authors also have added the income purchasing power indicator. This indicator is defined as the average value of the region's wages by the subsistence minimum.

The dataset presenting “Intergenerational Equity and Sustainability” is characterizes the social responsibility of the population towards future generations, which is determined by the size of the state (regional) debt of the entity, the demographic load (the ratio of dependents to the economically active population), carbon intensity and adjusted net savings (total economic, ecological and social index).

Institutional indicators of inclusive development used to generate data are divided into: education and skills development, as well as basic services and digital infrastructure (Table 1).

**Table 1:** Institutional indicators of the performance of inclusive development (components of human potential).

Institutional foundations	Foundation components	Indicators
Institutional indicators of human potential components		
Education and Skills Development	Access (7 indicators)	Coverage rates of school, pre-school, vocational education and supplementary education; Indicators of professional qualifications of the population; Accessibility and cost of higher and secondary education.
	Number (8 indicators)	Quality of the education system and satisfaction of the population. General availability of educational infrastructure; Workload of the teaching staff and cost of regions in education.
	Capital (4 indicators)	Overall state of the educational infrastructure and accessibility for people with limited mobility; Labor activity scope of graduates of higher educational institutions.
Basic services and digital infrastructure	Basic and digital infrastructure (14 indicators)	Indicators of the availability of mobile communications and the Internet; Indicators characterizing the degree of satisfaction of the population and their costs for transport infrastructure, housing and utilities, as well as indicators showing the degree of improvement of living conditions; Indicators of housing affordability, cost and rent.
	Services and infrastructure in the sphere of health, environment and law (12 indicators)	Life expectancy indicators; Drinking water availability and pollution factors; Reliability indicators of law enforcement agencies; Expenditures of the region on health care and general satisfaction of population with them; Overall condition of health infrastructure in the region.

Education and skills development reflects the general level of education in the region, its availability at all levels, as well as its effectiveness. It is characterized by such subgroups as access, quantity and capital (Table 1).

Basic services and digital infrastructure reflect the general condition of the entire infrastructure (healthcare, housing and utilities, transport system) and its availability to the public.

## 2 Experimental design, materials and methods

The database was formed after analyzing the information databases and processing requests received from official sources of state and regional statistics of socio-economic development.

At the first stage, descriptive approaches to statistical data analysis were applied, that is, the necessary key indicators for finding inclusive development were collected, which were then processed and calculated sequentially with the addition and adaptation of a number of indicators for each specific region of the Russian Federation.

At the second stage, indicators of the institutional bases of inclusive development were formed - education and infrastructure, which form the human potential components and were calculated by a number of indicators adapted to the peculiarities of the socio-economic development of the regions of the Russian Federation.

At the third stage, taxonomic analysis models were applied, that is, the values of inclusive growth indices of the regions of the Russian Federation were determined by key and institutional bases (education and infrastructure), they were calculated by collecting, analyzing and aggregating data on the socio-economic development of the Russian Federation and its regions, using formulas and methodologies proposed by experts of the World Economic Bank and the World Economic Forum for calculating the inclusive development index.

### 2.1 The essence and concept of inclusive growth: indicators of inclusive development performance

Global economic growth contributes to the gradual increase in the well-being of only a small part of the population, which causes increased inequalities throughout the world and the erosion of social cohesion in many developed and developing countries [2]. For this reason, the world community has started to stress the need to revise the traditional model of economic growth in order to put people's well-being in the spotlight, and in 2012 they have launched the inclusive growth initiative to develop a long-term policy strategy to support this new vision of growth [3]. This inclusive growth should be understood as a model of economic development aimed at creating and developing a balanced socio-economic system, taking into account social equality, environmental protection and natural resources [1]. In the same way, inclusive growth is understood as economic growth aimed at increasing wealth and reducing inequality through an open and equitable distribution of wealth among the population [2].

In January 2017, a report on the inclusive growth and development was presented at the World Economic Forum in Davos. In this report, the inclusive development index of more than 109 countries of the world was given, with the methodology of its assessment, as well as key indicators and the political and institutional foundations of inclusive development for which the index was formed. That report gave the final shape to the inclusive development policy [4]. Thanks to this report and the publicly available information from official statistical sites and databases, it has become possible to analyze, process, aggregate and structure the methodology for calculating inclusive growth and development of the regions of the Russian Federation and form the dataset. For the formation of the final dataset, open sources were used: the database of the Unified Interdepartmental Statistical Information System (UISIS); Federal State Statistics Service, Statistics Map of the Russian Federation, as well as the results of previous research on this topic.

Based on the World Bank methodology three groups of key indicators of inclusive development were identified and adapted (Fig. 1).

In Fig. 1, the authors added an indicator of Income purchasing power*. This indicator is defined as the average value of the region's wages per subsistence minimum. It was introduced to “smooth out” large values of the gross regional product in regions with an extremely low population, for example, the Kamchatka region, to show a more realistic standard of living among citizens.

Key performance indicators include not only the traditional indicator of gross domestic product, but also the most well-known intergovernmental indicators characterizing transformations in the living standards of the population.

**Fig. 2:**
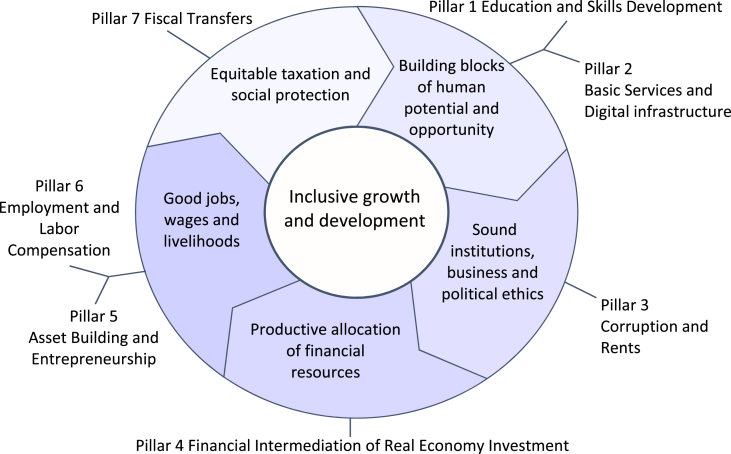
Inclusive growth and development circle [3].

Also, 8 institutional and political foundations of inclusive development were allocated, which are divided into 15 subgroups (Fig. 2).

The political and institutional foundations given in this picture represent the economic, social and political system of the state based on the institutions of society and government, whose development, within the context of inclusive development, contributes to the improvement of the population's quality of life, the increase of their incomes and security level [5].

All institutional and political foundations are important and play a certain role in inclusive development. However, in our opinion, human potential is the top priority, so inclusive development brings about an increase in human capabilities and growth of the human potential in socio-economic processes. On the other hand, inclusive development itself is possible with intensive human activity and using available economic opportunities [6]. Investment in human capital, especially in higher education, is one of the tools that can help stimulate inclusive growth [7], [8]. Accessibility of medical services, education, and other vital infrastructure, determine the quality of human capital [9]. Health care and education can also be used in assessing inclusive development, as indicators of the degree of equality of opportunities enjoyed by a country's population. This means that all members of society must be provided with the means to form the basic human possibilities that are the most important basis for social integration [10], [11].

The components of human potential are divided into groups: “Education and skills” and “Basic services and digital infrastructure”, which are necessary for human potential development. In order to assess human potential as an institutional foundation for inclusive development, indicators proposed by the world bank for these foundations were taken and supplemented according to the characteristics of the constituent entities of the Russian Federation (Table 1).

### 2.2 Methodology for assessing the index of inclusive development

The methodological basis of the research was constituted by scientific works of specialists in the sphere of economic growth and inclusive development. The methodology of the assessment of inclusive development was based on the methodology of the World Bank, adapted for regions of the Russian Federation.

At the first stage, the regions of the Russian Federation were ranked by their income and a group was selected for the dataset formation consisting of 26 regions of the Russian Federation.

At the second stage, the data necessary for finding key indicators of inclusive development were collected, processed, and calculated in sequence, with the addition and adaptation of a number of indicators specifically for Russian regions.

At the third stage, data were collected on the institutional foundations of inclusive development - education and infrastructure, which form the components of human potential, then the data were calculated for a number of indicators adapted to the particular socio-economic development of the regions of the Russian Federation.

At the fourth stage, the data obtained were grouped and presented as a table, which made it possible to construct the inclusive development index based on key and institutional foundations.

To carry out the assessment, the regions were divided into groups according to the per capita income factor – the ratio of the gross regional product to the total population in the territorial entity. All regions of Russia were classified into 4 groups:

1) With income levels of 500 thousand Russian rubles per year (10 regions);
2) With average income levels from 300 to 500 thousand rubles per year (26 regions was a group of regions);
3) With income levels s below the average from 200 to 300 thousand rubles a year (26 regions);
4) With a low income level up to 200 thousand rubles per year (20 regions).

To form a rating of inclusive development between regions based on key and institutional foundations, we used formulas (1), (2) and (3):

(1) $$6\times \frac{\left(\text{country}\;\text{score}\;-\;{\text{sample}}_{\min}\right)}{\left({\text{sample}}_{\max}-{\;\text{sample}}_{\min}\right)}+1$$

(2) $$-6\times \frac{\left(\text{country}\;\text{score}\;-\;{\text{sample}}_{\min}\right)}{\left({\text{sample}}_{\max}-\;{\text{sample}}_{\min}\right)}+7$$

(3) $${\text{category}}_{i}=\frac{\sum_{k=1}^{K}{\text{indicator}}_{k}}{K}$$

with *country score* being country-specific value of the indicator to form the dataset;

*Sample*_*min*_ and *sample*_*max*_ – minimum and maximum values of indicators for all the countries (regions) to form the dataset for a specific group of indicators.

(1), (2) are used to convert quantitative indicators by group into numerical values from 1 to 7, with 1 displaying the worst values, and 7 - the best values in the group. Formula (1) is used for indicators where a higher value is a positive factor, for example, life expectancy in a region. Formula (2) is used for indicators in which the higher the value, the more it negatively affects the socio-economic well-being of the entity, for example - the level of poverty. Formula (3) is used to sum all the indicators of a group, in order to form the general index of inclusive development or its intermediate values.

The informational basis of the dataset was formed by statistical data collected in regional and federal statistical information agencies - State Statistics and the Unified Interdepartmental Statistical Information System (UISIS) [12]. Data of the World Bank, the World Economic Forum and the Asian Development Bank on the issues of inclusive growth and development of the countries of the world as a whole and Russia in particular were also used. Statistical data on inequality - Standardized World Income Inequality Database (SWIID) [13].

### 2.3 A dataset on the inclusive development of regions of the Russian Federation by key foundations

To find the inclusive development index by key performance indicators, it is necessary to calculate three groups of values: inclusion, intergenerational equity and sustainability, and growth and development. We will calculate the group indicator - inclusiveness, namely the Gini coefficient (Table 2).

**Table 2:** Gini income inequality: The value of the indicator before and after conversion.

Russian Federation Region	Values over years											
	2012		2013		2014		2015		2016		2017	
	%	1–7	%	1–7	%	1–7	%	1–7	%	1–7	%	1–7
Krasnoyarsk Territory	42.5	3.05	42.3	3.02	40.8	3.52	39.8	3.84	40.0	2.93	39.2	3.53
Kamchatka Territory	38.1	6.39	38.1	6.06	37.0	6.83	36.1	6.76	34.5	7.00	34.4	7.00
Arkhangelsk Region	37.3	7.00	36.8	7.00	36.8	7.00	35.8	7.00	36.4	5.59	38.3	4.18
Murmansk Region	39.7	5.18	39.8	4.83	38.1	5.87	36.6	6.37	36.3	5.67	36.3	5.63
Tatarstan Republic	42.5	3.05	42.2	3.10	42.3	2.22	41.6	2.42	41.3	1.96	40.2	2.81
Leningrad Region	37.9	6.54	37.8	6.28	37.7	6.22	37.2	5.89	36.9	5.22	38.1	4.33
Belgorod Region	41.0	4.19	40.4	4.40	39.9	4.30	39.1	4.39	39.7	3.15	39.6	3.24
Khabarovsk Territory	38.9	5.78	39.0	5.41	38.6	5.43	37.9	5.34	38.5	4.04	38.6	3.96
Tomsk Region	39.1	5.63	38.9	5.48	37.8	6.13	36.5	6.45	35.4	6.33	34.5	6.93
Sverdlovsk Region	43.0	2.67	43.1	2.45	42.3	2.22	41.3	2.66	41.0	2.19	40.9	2.30
Moscow Region	42.0	3.43	41.7	3.46	40.6	3.70	39.2	4.32	39.7	3.15	39.0	3.67
Irkutsk Region	41.2	4.04	40.9	4.04	38.6	5.43	37.5	5.66	37.2	5.00	37.1	5.05
Perm Territory	42.8	2.82	43.0	2.52	42.7	1.87	42.4	1.79	41.2	2.04	40.7	2.45
Orenburg Region	39.1	5.63	39.2	5.27	39.3	4.83	38.3	5.03	37.9	4.48	38.4	4.11
Samara Region	44.2	1.76	44.1	1.72	42.2	2.30	41.4	2.58	38.2	4.26	37.9	4.47
Vologda Region	37.7	6.70	37.7	6.35	37.3	6.57	36.5	6.45	37.0	5.15	35.8	5.99
Primorye Territory	39.2	5.56	38.4	5.84	38.9	5.17	38.4	4.95	38.0	4.41	38.5	4.04
Novgorod Region	40.6	4.49	39.8	4.83	39.4	4.74	37.9	5.34	37.1	5.07	36.6	5.41
Novosibirsk Region	41.5	3.81	41.0	3.96	38.5	5.52	36.9	6.13	37.5	4.78	36.5	5.48
Krasnodar Territory	42.0	3.43	42.3	3.02	42.4	2.13	41.4	2.58	41.5	1.81	40.9	2.30
Kaliningrad Region	39.2	5.56	38.6	5.70	38.6	5.43	37.1	5.97	36.5	5.52	36.2	5.70
Lipetsk Region	39.7	5.18	40.0	4.69	39.8	4.39	38.8	4.63	38.9	3.74	38.8	3.82
Kaluga Region	40.1	4.87	39.6	4.98	38.9	5.17	37.7	5.50	37.6	4.70	37.1	5.05
Yaroslavl Region	39.2	5.56	39.3	5.19	39.4	4.74	38.7	4.71	38.5	4.04	37.3	4.90
Bashkortostan Republic	42.8	2.82	42.8	2.66	42.5	2.04	41.4	2.58	41.6	1.74	41.7	1.72
Nizhni Novgorod Region	40.5	4.57	41.2	3.82	41.2	3.17	40.3	3.45	40.3	2.70	39.9	3.02

In this form quantitative indicators appear in all the regions, and later on a consistent aggregation of indicators is made for each individual group and for the regions, after which an index is formed for the three main groups - growth and development, inclusion, intergenerational equity and sustainability (Table 3). The indicators of these three groups are added using formula (3), and the final index of inclusive development is formed (Table 4).

**Table 3:** The final index of inclusive growth and development of the regions of the Russian Federation (26 regions) (from 1 to 7) [1].

Regions		Final Index of Inclusive Development						
		2012	2013	2014	2015	2016	2017	Mean value, as a whole
1	Moscow Region	4.29	4.20	4.00	3.94	4.23	4.13	4.13
2	Tatarstan Republic	4.10	4.11	3.88	3.87	3.78	4.22	4.00
3	Kamchatka Territory	4.00	3.90	3.90	4.04	3.86	3.84	3.92
4	Murmansk Region	3.66	3.92	3.63	3.97	3.91	3.77	3.81
5	Leningrad Region	4.09	3.79	3.44	3.54	3.48	3.55	3.65
6	Belgorod Region	3.81	3.74	3.60	3.48	3.43	3.82	3.65
7	Arkhangelsk Region	3.61	3.59	3.27	3.49	3.04	3.50	3.42
8	Sverdlovsk Region	3.49	3.52	3.17	3.12	2.96	3.40	3.28
9	Krasnoyarsk Territory	3.30	3.23	3.38	3.36	3.05	3.20	3.25
10	Krasnodar Territory	3.14	3.27	3.06	3.18	3.25	3.54	3.24
11	Samara Region	3.14	3.26	3.08	3.17	2.85	3.12	3.11
12	Khabarovsk Territory	3.07	3.17	3.06	3.07	3.08	3.13	3.10
13	Kaluga Region	3.22	2.99	2.96	2.80	2.91	3.27	3.03
14	Nizhni Novgorod Region	2.99	2.95	2.99	2.93	2.90	2.95	2.95
15	Primorye Territory	2.98	2.91	2.80	2.99	2.93	3.01	2.94
16	Kaliningrad Region	3.14	2.92	3.08	2.68	2.73	2.92	2.91
17	Lipetsk Region	2.95	2.75	2.84	2.84	2.71	2.79	2.81
18	Yaroslavl Region	2.78	2.76	2.93	2.96	2.60	2.84	2.81
19	Perm Territory	3.09	2.87	2.61	2.74	2.46	2.90	2.78
20	Bashkortostan Republic	2.97	2.71	2.45	2.52	2.52	2.98	2.69
21	Novosibirsk Region	2.76	2.72	2.57	2.61	2.64	2.80	2.68
22	Novgorod Region	2.69	2.68	2.52	2.71	2.51	2.63	2.62
23	Tomsk Region	2.68	2.69	2.66	2.54	2.48	2.57	2.60
24	Orenburg Region	2.54	2.84	2.62	2.40	2.13	2.64	2.53
25	Vologda Region	2.58	2.39	2.25	2.49	2.58	2.64	2.49
26	Irkutsk Region	2.38	2.37	2.22	2.34	2.22	2.34	2.31
Mean values, over years		3.21	3.16	3.04	3.07	2.97	3.17	3.10

**Table 4:** The total index for the group - the components of human potential (from 1 to 7).

Regions		Human Potential Value						
		2012	2013	2014	2015	2016	2017	Mean value, as a whole
1	Krasnodar Territory	3.08	3.13	3.27	3.34	4.79	3.30	3.48
2	Moscow Region	3.36	3.20	3.49	3.71	3.17	3.90	3.47
3	Tatarstan Republic	3.16	3.25	3.30	3.37	3.86	3.70	3.44
4	Sverdlovsk Region	3.18	3.57	3.29	3.23	3.13	3.44	3.31
5	Belgorod Region	2.66	2.38	3.15	3.03	3.61	2.98	2.97
6	Nizhni Novgorod Region	2.87	2.58	2.84	2.88	3.17	3.06	2.90
7	Krasnoyarsk Territory	2.86	2.70	3.05	3.06	2.58	2.97	2.87
8	Bashkortostan Republic	2.73	2.67	2.90	2.90	2.81	2.85	2.81
9	Perm Territory	2.71	2.76	2.77	2.97	2.67	2.87	2.79
10	Orenburg Region	2.62	2.65	2.81	2.84	2.76	2.71	2.73
11	Kamchatka Territory	2.78	3.06	2.67	2.94	1.70	2.85	2.67
12	Samara Region	2.56	2.79	2.59	2.74	2.54	2.72	2.66
13	Murmansk Region	2.63	2.31	2.88	2.91	2.16	2.90	2.63
14	Novosibirsk Region	2.59	2.45	2.95	2.64	2.24	2.83	2.62
15	Yaroslavl Region	2.41	2.47	2.87	3.00	2.54	2.42	2.62
16	Primorye Territory	2.67	2.68	2.66	2.51	2.44	2.56	2.59
17	Kaluga Region	2.38	2.29	2.79	2.68	2.73	2.56	2.57
18	Lipetsk Region	2.39	2.47	2.45	2.44	2.90	2.58	2.54
19	Leningrad Region	2.39	2.50	2.51	2.65	2.58	2.53	2.53
20	Tomsk Region	2.61	2.59	2.63	2.67	1.90	2.69	2.51
21	Novgorod Region	2.58	2.22	2.69	2.70	2.41	2.06	2.45
22	Arkhangelsk Region	2.33	2.36	2.68	2.57	2.11	2.40	2.41
23	Khabarovsk Territory	2.32	2.02	2.53	2.54	2.70	2.28	2.40
24	Kaliningrad Region	2.30	2.50	2.59	2.56	1.83	2.26	2.34
25	Irkutsk Region	2.26	2.25	2.37	2.43	1.27	2.20	2.13
26	Vologda Region	2.06	1.85	1.93	2.13	2.01	2.41	2.07
Mean values, over years		2.63	2.60	2.79	2.82	2.64	2.77	2.71

The final index of inclusive development, formed by key performance indicators divided into three groups, characterizes the overall performance of state and regional policies aimed at increasing the well-being of the population, developing human capital and the level of environmental preservation for future generations. According to the final index, the inclusive development in most regions worsens from year to year, which is due to a number of reasons: the growing poverty level in many regions, the presence of government debt in most entities, the constantly deteriorating environmental situation and low incomes of the population.

### 2.4 A dataset on the institutional foundations for the inclusive development of the regions of the Russian Federation

In addition to key indicators of the performance of inclusive development, institutional and political indicators of inclusive development are identified, whose role is no less important because they show the strengths and weaknesses of countries and regions by specific sectors: human capital, politics, economics and finance, the environment. In our work we will consider the components of human potential as one of the institutional foundations for inclusive development, the components consisting of two subgroups: education, and basic services with digital infrastructure. These components are responsible for developing the human potential necessary for productive use of the existing economic opportunities by all citizens [6], [14]. The calculation of this group of indicators is similar to the method of finding key indicators, using (1), (2), (3) (Table 4).

In Table 4, the data set of the human potential components is ranked in decreasing order of averages - from the best (Krasnodar Territory) to the worst (Vologda Region). In the regions of Russia, as well as in the country as a whole, traditionally there is a strong human capital, which is the legacy of the Soviet Union, however, in the modern time it begins to lose ground on some points. Most regions have a high coverage of children with preschool education, over half of the children are engaged in this or that additional education. Population satisfaction with the quality of education leaves much to be desired and amounts to just a little over 50% in most regions. In the regions of the Russian Federation, there is a marked increase in the availability of infrastructure for sports and the availability of medical services. The population of most regions have constant access to high-quality clean drinking water (over 90%). The share of the population actively using the worldwide network is growing, including the number of goods and services ordered via the Internet. The share of substandard housing is decreasing. In general, the educational and infrastructural foundations of human potential in the regions of Russia are comparable to the national mean values. There is a large unused potential, but its implementation requires proper administrative changes. All this is important because the macroeconomic stability, human capital and structural changes are key determinants of inclusive growth in developing and developed countries, therefore human potential is also one of the most important conditions for inclusive development, and, therefore, it is important for the regions of Russia [15].

In general, the dataset presented reflect the indicators of inclusive development not only in the regions of the Russian Federation, but throughout the country as a whole. Thus, for example, while the Russian Federation ranked 13th in the index of inclusive development in 2017, by the end of 2018 it has moved to the 19th position, which is accounted for by the deterioration of the situation in its regions [4]. However, in the Russian Federation there is a good human potential that is not sufficiently implemented, and if used properly, it may become a zone of growth for inclusive development [16], [17]. All the data obtained indicate the importance of the urgent formation of the inclusive development policy based on the experience of countries of the world that have applied similar strategies, taking into account human potential as an institutional foundation for inclusive growth.
