# Supplementary material for: Inclusive growth: A dataset on key and institutional foundations for inclusive development of Russian regions
Source: Data Brief. 2019 Mar 20;23:103864. doi: 10.1016/j.dib.2019.103864 (PMC6660622; doi:10.1016/j.dib.2019.103864)
Supplement: Multimedia component 1 [file mmc1.docx]

Dear editor

Article “Inclusive Growth: A Dataset on Key and Institutional Foundations for Inclusive Development of Russian Regions” does not contain the conflict of interests

**Conflict of interest**

None.

With best regards and best wishes, the team of authors of the article “Inclusive Growth: A Dataset on Key and Institutional Foundations for Inclusive Development of Russian Regions”.
